# Prognostic factors of successful on-purpose tumor biopsies in metastatic cancer patients included in the SHIVA prospective clinical trial

**DOI:** 10.18632/oncotarget.12051

**Published:** 2016-09-15

**Authors:** Emilie Desportes, Mathilde Wagner, Maud Kamal, Anne Vincent Salomon, Gabrielle Deniziaut, Gaëlle Pierron, Etienne Rouleau, Thomas Jouffroy, Christophe Le Tourneau, Xavier Paoletti, Vincent Servois

**Affiliations:** ^1^ Departement of Radiology and Nuclear Medecine, Institut Curie, Paris, France; ^2^ Department of Medical Oncology, Institut Curie, Paris, France; ^3^ Department of Genetic, Institut Curie, Paris, France; ^4^ Department of Pathology, Institut Curie, Paris, France; ^5^ Department of Biostatistics and Epidemiology & INSERM U1018 oncostat, Gustave Roussy Cancer Campus, Villejuif, France; ^6^ Department of ENT Surgery, Institut Curie, Paris, France

**Keywords:** cellularity, biopsy, targeted therapies, CT-guided biopsy, precision medicine

## Abstract

**PURPOSE:**

To identify patient/tumor characteristics associated with success of biopsy in patients who received multiple lines of chemotherapy.

**METHODS:**

Patients with refractory cancer from our center, who were included in a prospective randomized phase II trial comparing targeted therapies based on molecular profile of tumors versus conventional chemotherapy, were retrospectively included in this IRB-approved study. All patients had a biopsy of a tumor lesion performed during surgery, or using CT/palpation/endoscopic guidance. A biopsy was considered successful if the neoplastic cellularity was greater than 30%. Primary lesion, size and location of biopsied lesion, on-going chemotherapy and the differential attenuation between non-enhanced and venous phase (HU) for CT-guided biopsied lesions were recorded.

**RESULTS:**

228 patients (age=59±15yo; M/F=1.9) were included. One hundred and sixty biopsies (72%) of the 221 biopsies performed were successful. Prognostic factors of biopsy success were: no ongoing chemotherapy, surgical or palpation-guided biopsy, lymph nodes/soft tissue location(*P* <0.01). Among the 221 performed biopsies, 122 (55%) were performed using CT guidance and 82 (67%) were successful. In this subgroup, biopsied lesions located in lymph nodes/soft tissue were associated with a higher success rate while lung location was associated with failure (*P* <0.01). The mean differential attenuation was significantly higher in lesions with a successful biopsy (*P* <0.001).

**CONCLUSION:**

Success of biopsy was less frequent with CT guidance than with surgical or palpation-guided biopsy and was higher in soft tissues and lymph nodes than that in visceral metastasis. Ongoing chemotherapy decreased tumor cell content and consequently the success of the biopsy samples for molecular profiling.

## INTRODUCTION

Nowadays, cancer treatments are chosen according to tumor factors such as primary tumor diagnosis or site, nodal status, metastatic status, histologic subtype or molecular alterations such as hormone receptor status in breast cancer patients, in addition to patient factors such as performance status, age, and comorbidities [1, 2]. Targeted therapies have emerged and were shown to provide antitumor efficacy only when their targets are present, such as trastuzumab in HER2-positive breast cancer patients or imatinib with Philadelphia chromosome in chronic myeloid leukemia [3, 4]. One of the current burning questions is whether targeted therapies should be given based on molecular alterations in a histology-agnostic way. This approach, often named precision medicine approach, consists of evaluating the molecular composition of evolving tumors and detecting biomarkers for new patient stratification [5, 6]. The molecular profile of each tumor must be analyzed, and targeted therapy can be recommended based on genomic characterization, with the aim of maximizing treatment efficacy, while minimizing undesirable side effects. Some of these therapies, consisting of small molecule inhibitors or antibodies, have already been proven to induce significant clinical responses in different refractory metastatic tumors, especially in breast and lung cancers [5–9] but also in other tumors like GIST or leukemia [10, 11].

Molecular profiling requires reassessment of tumor tissue and the collection of samples using biopsies with specific characteristics. First, molecular profiling requires a neoplastic cellularity in the tumor sample adequate to the limit of detection related to the technique used (Comparative Genomic Hybridation-array, Next-Generation Sequencing). To reduce the risk of false negatives, some studies have chosen a threshold greater than 50% of neoplastic cells in biopsies from which samples qualified for further genomic testing [12]. The approaches for copy-number alteration often need up to 20% tumor cells in samples [13]. Secondly, these biopsies for molecular profiling are often performed in patients who present refractory metastatic solid tumor after many lines of chemotherapy. The history of previous chemotherapy often causes structural changes to the tumors like necrosis and fibrosis [14] which might consequently decrease tumor samples' neoplastic cellularity. Therefore, the success rate can be defined as the number of biopsies with at least 30% neoplastic cells.

There are four main ways to collect tumor samples: surgical, palpation-guided biopsy, endoscopy and radiological-guided biopsies. Palpation-guided biopsy is rarely feasible due to deep visceral localization of metastasis, while surgery and bronchoscopy in some cases need general anesthesia. Many studies showed that CT-guided needle biopsy enables the acquisition of sufficient tumor material for molecular analyses, while being simple and less invasive than surgery, especially in lung cancer [15, 16].

Some teams assessed the feasibility, the safety and/or the success of CT-guided biopsies defined as completion of the biopsy procedure with pathologic confirmation of malignant lesion in the context of targeted therapies [15–17]. However, very few studies assessed the causes of biopsy failure defined with the neoplastic cellularity whatever the type of cancer [18].

Therefore, the purpose of this study was to identify factors associated with cellularity and biopsy success rates, particularly in CT-guided biopsies, in metastatic patients who received multiple lines of chemotherapy.

## MATERIALS AND METHODS

### Study design

This study is a retrospective analysis of the data of the clinical trial SHIVA which was a multicenter, randomized controlled phase 2 trial of molecularly targeted agents based on tumor molecular profiling *versus* treatment in patients with refractory cancer [19]. The eligibility criteria of SHIVA trial were recurrent or refractory solid tumour, measurable in accordance with RECIST 1.1 and with a tumoral site accessible for a biopsy or resection in patients older than 18 years. A tumor sample obtained by biopsy or surgery was required for the inclusion in the SHIVA trial to obtain a molecular profiling in order to lead the targeted therapies.

The study protocol was approved by ethics committee and the institutional review board ; written consent was obtained from all patients. The current study was retrospective and included all patients of the SHIVA trial treated in the Institute Curie (Paris) who underwent biopsy between September 2012 and August 2014. Finally, 228 patients were included, 78 men and 150 women with a mean age of 58,9 ± 13.9 y and a mean BMI of 23.6 ± 3.9 kg/m ^2^.

### Biopsy procedure

A biopsy of the primary lesion or metastasis (the most accessible lesion, except cerebral and osseous metastasis) was required to obtain a tumor sample for molecular analyses. All patients were discussed in a multidisciplinary board (including oncologists, surgeons, radiologists and pathologists) to decide the technique of the biopsy. Biopsies were performed using four different techniques: 1/ Surgical biopsies (incisional and excisional biopsies), performed by surgeons from the gynecology and ENT departments; 2/ CT-guided biopsies performed by a radiologist; 3/ Palpation-guided biopsies, performed by a pathologist from the pathology department or 4/ Endoscopic biopsies performed by a pneumologist or a gastroenterologist. There were no one-site cytopathologist to immediately assess the presence of viable tumor in the specimen.

The CT-guided biopsies were performed with a GE CT system (Lightspeed VCT, General Electric healthcare, Milwaukee), using an automated 17- or 19-gauge co-axial system (SuperCore ^TM^ Argon Medical Devices, Athens) and by three intervention-trained radiologists, who had performed more than 5000, 1000 and 400 imaging-guided biopsy procedures respectively. The palpation-guided biopsies were performed using 16- or 14-gauge needles. The endoscopic biopsies were performed using 2.2 mm diameter forceps. No fine needle aspiration biopsies were used.

### Pathology analysis

At least four tumor samples were required for each patient enrolled in the trial. One sample was formalin-fixed and paraffin-embedded for diagnostic confirmation, and hormone receptors expression analyses. The other biopsies were fresh frozen. One of them was used for DNA extraction for molecular analyses and the remaining was stored for possible ancillary studies. The tumor cellularity was performed on the formalin-fixed paraffin-embedded after staining with hematein-eosin-safran and the frozen specimens.

Pathologists of our department analyzed the tissue samples. One pathologist with more of 30 years of experience visually estimated and quantified in percent the presence of tumor cells to assess the neoplastic cellularity. Necrosis was estimated semi-quantitatively with a 4-point scale (0 = absence, 1 = low, 2 = medium, 3 = high). A retrospective review by the same pathologist was specifically performed to analyze more precisely the biopsies failures cases and quantify neoplastic cellularity, necrosis and fibrosis of the samples. Based on the literature data and our gene laboratory experience, a biopsy was considered to be successful if cellularity was higher than 30% [8, 12, 13]

### Data analysis

Each of the following clinical data was collected: age, gender, weight, BMI, histological type of cancer, number of previous chemotherapy lines, ongoing chemotherapy during biopsy.

An enhanced-CT scan was performed less than one month before biopsy for each patient which underwent a CT-guided biopsy. A radiologist with 4 years of experience blinded to the clinical data reviewed the CT images in random order on a PACS workstation to collect target lesion characteristics (Carestream Health, Version 11.4, Mountain View, CA). Patients' identities were removed from the images. CT data included the biopsied organ, the target lesion size (largest diameter) and the enhancement of the lesion. A 20 mm diameter ROI was drawn at the distal end of the co-axial needle in the axis of the cutting needle on the non-enhanced image. The ROI was then copied and pasted onto the enhanced image, with care to match anatomical location between the enhanced and unenhanced images. The attenuation (mean HU in the ROI) of the lesion on the non-enhanced and enhanced images was recorded and the attenuation variation ΔHU (mean HU of ROI after contrast minus mean HU of ROI before contrast) was computed for each biopsied lesion.

Biopsy characteristics including the needle size and the size of biopsied lesion were collected. The number of collected samples of CT-guided biopsies was set at four.

### Statistical analysis

Descriptive results are presented as mean ± standard deviation or median (ranges) for quantitative data and as frequency (percentage of cases) for categorical variables. The correlation between quantitative approximately normal variables (age, BMI, weight) and cellularity was analyzed using Pearson correlation coefficient. The association between categorical variables and cellularity was explored in an ANOVA using Fisher global test, followed by a Tukey test in case of significant difference in order to adjust for multiple testing. The association between categorical variables and biopsy success (defined as a neoplastic cellularity greater than 30%) was performed using Chi-square or Fischer exact tests as appropriate. The same analysis was repeated on the subgroup of patients with CT-guided biopsy. The prognostic value of the ΔHU to identify successful biopsies was assessed by the receiver operating characteristic (ROC) curve. Cutoff values were further derived to by maximizing the Youden index (sum of the sensitivity plus specificity minus one).

Tests were two-sided, with a level of significance set at *P* < 0.05. All analyses were performed using Statistical Analysis Software (SAS) (version 9.4, SAS Insitute Inc., Cary, NC).

## RESULTS

### Population and tumor

**Figure 1:**
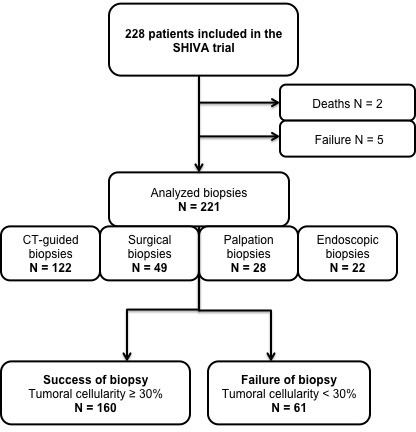
Flow chart of the study

The flowchart of the study is presented in Figure 1. Characteristics of the included population and the tumors are presented in Table 1. The most frequent histological subtype was adenocarcinoma (131/228, 59%). The most frequent locations for the primary lesion were breast, lung and ENT region, in 18%, 17% and 17% of patients, respectively. Forty-five patients (44%) had no chemotherapy when the biopsy was performed, with a mean stop of 69 ± 89 days before the biopsy.

**Table 1:** Patients and lesions characteristics

	Total population (*N*= 221 patients)	Population with CT-guided biopsy (*N* = 122)
**Sex** ^b^		
• Male	75 (34%)	45 (37%)
• Female	146 (66%)	77 (63%)
**Age**^a^		
• Male	58.9 ± 13.9	56.8 ± 15.0
• Female	59.4 ± 13.5	60.1 ± 13.4
**Weight** (Kg)^a^	66.9 ± 15.4	66.7 ± 12.5
**BMI**^a^	23.6 ± 4	23.5 ± 3.6
**Cancer** ^b^		
• Histology		
– Adenocarcinoma	131 (59%)	76 (62%)
– Epidermoid carcinoma	37 (17%)	13 (11%)
– Sarcoma	17 (8%)	12 (10%)
– Others	36 (16%)	21 (17%)
• Primary location		
– Lung	38 (17%)	23 (19%)
– Breast	40 (18%)	20 (16%)
– ENT	24 (11%)	8 (7%)
– Ovaries	24 (11%)	16 (13%)
– Colon	15 (7%)	9 (7%)
– Uterus	15 (7%)	5 (4%)
– Stomach	5 (2%)	3 (2%)
– Pancreas	7 (3%)	6 (5%)
– Bladder	6 (3%)	4 (3%)
– Bone	8 (4%)	6 (5%)
– Eye	4 (2%)	2 (2%)
– Others	27 (12%)	16 (13%)
– Unknown	8 (4%)	4 (3%)
**Treatment**		
• Ongoing chemotherapy^b^	145 (66%)	45 (37%)
• Median number of lines of previous		
– 0	15 (7%)	6 (5%)
– 1-4	142 (64%)	75 (61%)
– ≥ 5	64 (29%)	41 (34%)
**Guidance**^b^		
• Surgery	49 (22%)	-
• Endoscopy	22 (10%)	-
• CT	122 (55%)	-
• Palpation biopsy	28 (13%)	-
**Site** ^b^		
• Liver	65 (29%)	63 (51%)
• Nodes	37 (17%)	8 (7%)
• Soft tissue	34 (15%)	11 (9%)
• Lung	26 (12%)	24 (20%)
• Bronchi	15 (7%)	
• Peritoneum	12 (6%)	8 (7%)
• Digestive system	7 (3%)	
• Vagina	6 (3%)	
• Adrenal gland	5 (2%)	4 (3%)
• Breast	4 (2%)	
• ENT	3 (1%)	
• Uterus	3 (1%)	
• Others	4 (2%)	4 (3%)
**Size of the biopsied lesion**^a^ (available for 120 patients)	-	46.7 (23.0)

a data are presented as mean ± standard deviation

b data are presented as number of cases (percentage of cases)

### Biopsies

For seven patients, no biopsy was performed due to death before the biopsy (two patients), to a technical issue making it not possible to perform a biopsy (four patients) or a mere failure of the biopsy (one patient). Those patients were excluded from the analysis, leaving a total of 221 subjects.

Biopsies characteristics are presented in Table 1. More than half of the biopsies were performed using CT guidance (*n* = 122). An 18-gauge needle was used in 103 patients (84%) including 10 lung biopsies and a 20-gauge needle in 19 patients (16%) including 14 lung biopsies. The more frequent site of the biopsy was the liver (65/228, 29%) followed by lymph nodes (37/228, 17%). Among the 221 performed biopsies, 160 (72%) were considered as successful. The 28% of the patients that did not obtain a successful biopsy were excluded from the initial SHIVA trial and received no targeted-therapies.

### Complications of biopsy

There were no complications for palpation-guided biopsy and endoscopic biopsies. Post-procedural complications occurred in five patients who underwent CT-guided biopsy. The complications consisted of pneumothorax (5/24 (21%) of chest CT-guided biopsies). Chest tube placement was required in one of five patients with pneumothorax. In the remaining four patients, pneumothorax was small and asymptomatic and resolved spontaneously. Post-surgical complications occurred in five patients (10%) who underwent pelvic amputation (three cases) and retroperitoneal lymphadenectomy (two cases). One infected pelvic hematoma and one refractory chylous ascites required surgical reintervention.

### Prognostic factors of neoplastic cellularity and biopsy success in all population

Prognostic factors of neoplastic cellularity are presented in Table 2. Patients with ongoing chemotherapy had a neoplastic cellularity significantly lower (41%) than patients without ongoing chemotherapy (52%) (*P* = 0.015). There was no significant association between number of lines of chemotherapy and cellularity. There was a significant association between histological sub-types and cellularity (*P* = 0.002). The histological sub-type with the highest neoplastic cellularity was sarcoma (mean cellularity = 67%). The biopsy type with the highest mean cellularity percentage was palpation-guided biopsy (mean cellularity = 59.3%) and the one with the lowest cellularity was bronchoscopy (mean cellularity = 28.4%). The highest mean cellularity was found in lymph nodes and soft tissues (mean cellularity = 62.6% and 61.6%, respectively) while the lowest one was found in lung and bronchi (mean cellularity = 31.6 and 16.7%, respectively).

**Table 2:** Prognostic factors of cellularity (significant p values are in bold)

	WHOLE POPULATION			CT-GUIDED BIOPSIES		
	Pearson correlation coefficient R	Mean cellularity ± SD	*P* value	Pearson correlation coefficient R	Mean cellularity ± SD	*P* value
**Age**	−0.03	_	0.73	−0.03	_	0.76
**BMI**	0.08	_	0.26	0.09	_	0.33
**Weight**	0.13	_	0.06	0.2	_	**0.025**
**Ongoing chemotherapy**			**0.015**			0.068
* Yes		41.3 ± 31.9			38.8 ± 32.3	
* No		51.6 ± 28.3			49.3 ± 29.1	
**Nb of lines of chemotherapy**			0.537			0.429
* 0	_	42.3 ± 33.5		_	32.5± 39.2	
* 1-4	_	47.4 ± 31.0		_	44.5 ± 32.0	
* ≥ 5	_	51.0 ± 26.6		_	49.0 ± 26.6	
**Histology**			**0.002**			0.009
* Adenocarcinoma	_	43.9 ± 27.5		_	39.9 ± 28.2	
* Epidermoid carcinoma	_	44.5 ± 33.6		_	40.4 ± 37.1	
* Sarcoma	_	67.7 ± 29.9		_	66.7 ± 26.5	
* Others	_	57.9 ± 29.7		_	56.2 ± 30.9	
**Primary location**			**0.026**			0.055
**Guidance**			**< 0.0001**	-	-	-
* Surgery	_	57.9 ± 22.8				
* Endoscopy	_	28.4 ± 31.9				
* CT	_	45.1 ± 30.4				
* Palpation biopsy	_	59.3 ± 27.5				
**Localization of biopsy**			**< 0.0001**			**0.04**
* Node	_	62.6 ± 22.5		_	60.0 ± 25.6	
* Soft tissue	_	61.8 ± 27.1		_	60.9 ± 28.1	
* Other	_	58.9 ± 21.7		_	65 ± 12.9	
* Liver	_	47.4 ± 27.8		_	47.3 ± 28.1	
* Digestive	_	47.1 ± 28.1				
* Vagina	_	45.8 ± 23.8				
* Adrenal glands	_	38 ± 34.9		_	47.5 ± 32.0	
* Peritoneum	_	36.7 ± 19.1			34.0 ± 20.4	
* Lung	_	31.6 ± 36.6		_	27.9 ± 35.7	
* Bronchi	_	16.7 ± 24.7				

Prognostic factors of biopsy success are reported in Table 3. Successful biopsies with more than 30% cancer cells occurred in 160 of the 221 biopsies (72%). Similarly to the findings on neoplastic cellularity, prognostic factors of biopsy success were: no ongoing chemotherapy (47/76, 62%), palpation-guided biopsy (25/28, 90%), localization of biopsy in lymph nodes (34/37, 92%) and in soft tissue (31/34, 91%).

**Table 3:** Prognostic factors of biopsy success (significant *p* values are in bold)

	WHOLE POPULATION			CT-GUIDED BIOPSIES		
	Success *N* = 160	Failure *N* = 61	*P* value	Success *N* = 82	Failure *N* = 40	*P* value
**Age**	59.2 ± 13.3	59.2 ± 12.3	0.98	58.9 ± 14.6	58.8 ± 12.9	0.95
**BMI**	23.8 ± 23.1	23.3 ± 22.6	0.33	24.0 ± 3.9	22.7 ± 2.7	0.07
**Weight**	67.6 ± 14.3	65.3 ± 11.2	0.2	68.7 ± 12.8	62.7 ± 10.9	**0.01**
**Ongoing chemotherapy**^b^			**0.01**			0.068
* Yes	47 (62%)	29 (38%)		26 (58%)	19 (27%)	
* No	113 (78%)	32 (22%)		56 (73%)	21 (42%)	
**Nb of lines of chemotherapy**^b^			0.149			0.102
* 0	8 (53%)	7 (47%)		2 (33%)	4 (67%)	
* 1-4	102 (61%)	40 (39%)		49 (65%)	26 (35%)	
* ≥ 5	50 (78%)	14 (22%)		31 (76%)	10 (24%)	
**Histology**^b^			0.057			**0.041**
* Adenocarcinoma	93 (71%)	38 (29%)		48 (63%)	28 (37%)	
* Epidermoid carcinoma	22 (60%)	15 (40%)		6 (46%)	7 (54%)	
* Sarcoma	15 (88%)	2 (12%)		11 (92%)	1 (8%)	
* Others	30 (83%)	6 (17%)		17 (81%)	4 (19%)	
**Primary location**	-	-	0.140			**0.046**
**Guidance**^b^			**< 0.0001**			
* Surgery	43 (88%)	6 (12%)				
* Endoscopy	10 (45%)	12 (54%)				
* CT	82 (67%)	40 (33%)				
* Palpation biopsy	25 (90%)	3 (11%)				
**Localization of biopsy**^b^			**0.0001**			**0.001**
* Liver	48 (74%)	17 (26%)		46 (73%)	17 (27%)	
* Nodes	34 (92%)	3 (8%)		7 (88%)	1 (12%)	
* Soft tissue	31 (91%)	3 (9%)		10 (91%)	1 (9%)	
* Lung	10 (38%)	16 (62%)		8 (33%)	16 (67%)	
* Bronchi	4 (27%)	11 (73%)				

### Prognostic factors of neoplastic cellularity and biopsy success in the subgroup of patients with CT guided biopsy

Prognostic factors of neoplastic cellularity in CT-guided biopsy population are presented in Table 2. As in the whole population of the study, the cellularity was associated with the histological sub-type of the tumor (sarcoma, mean cellularity = 68%, *P* = 0.009). Localization of biopsy was significantly associated with the highest neoplastic cellularity (*P* = 0.04) for soft tissue (mean cellularity = 61%) and lymph nodes (mean cellularity = 60%), while lung biopsy had the lowest (mean cellularity = 28%). Unlike in the whole population, the cellularity was not significantly associated with the absence or the presence of ongoing chemotherapy, but there was a trend with lower cellularity in cases of ongoing chemotherapy (*P* = 0.068).

Prognostic factors of biopsy success in CT guided biopsy population were reported in Table 3. Biopsy success occurred in 82 of 122 biopsies (67%). One of the prognostic factor of biopsy success was localization of biopsy (*P* = 0.002) in soft tissue (10/11, 91%) and lymph nodes (7/8, 88%), while failure occurred mostly in lung (16/24, 67%). The rate of success was also significantly higher in case of sarcoma (11/12, 92%), than in other histologic sub-types. Surprisingly, a lower weight was associated with a biopsy success.

**Figure 2:**
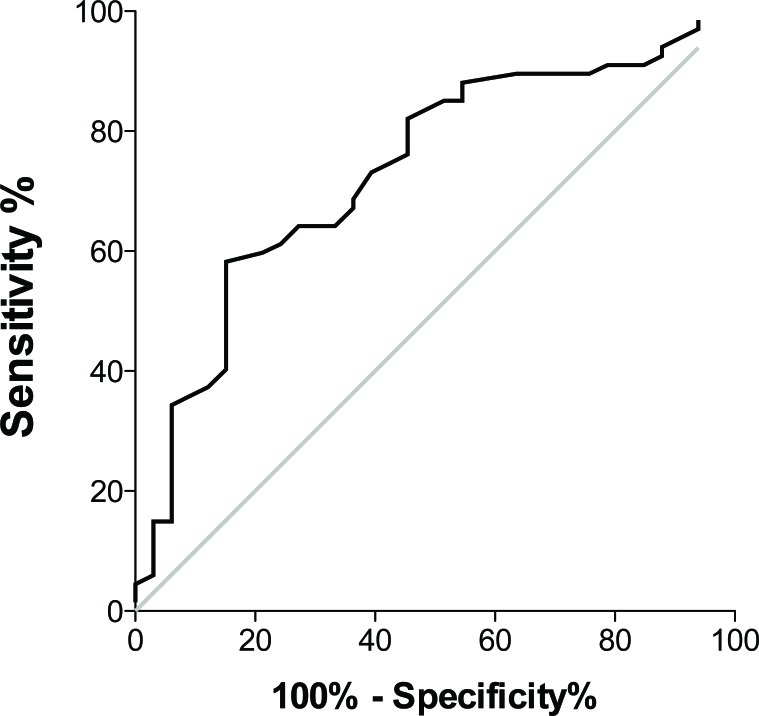
ROC curves of the attenuation variation for differentiating biopsy failure and success The AUROC was 0.73 ± 0.05 (95%CI 0.63 ; 0.84, *P* = 0.00017).

The mean attenuation variation was significantly higher in lesions with a biopsy success (33.6 HU *vs*. 19.1 HU, *P* < 0.001) (*P* = 0.008 in the multivariate analysis). The AUROC was 0.73 ± 0.05 (95%CI 0.63; 0.84, *P* < 0.001) (Figure 2). The optimal cutoff was 23 HU, with a sensitivity of 87% and a specificity of 45% for a biopsy success. The biopsied lesion size was not different depending on the success/failure.

For CT-guided biopsies failures (*n* = 40), an 18-gauge needle was used in 32 (80%) patients including eight lung biopsies and a 20-gauge needle in eight (20%) patients including eight lung biopsies. Among the 17 failures linked to the absence of tumor or insufficient tumor tissue, ten were performed using an 18-gauge needle (six liver biopsies, four lung biopsies and one soft tissue biopsy) and six using a 20-gauge needle (six lung biopsies). CT-guided core needle lung biopsies represent 59% (10/17) of this type of failure.

**Figure 3:**
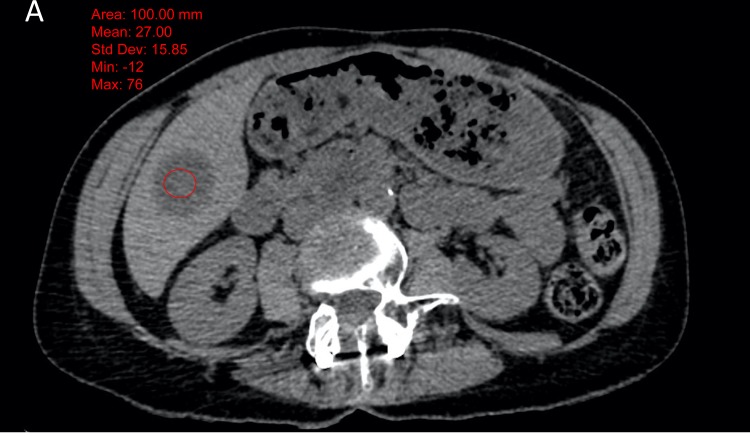

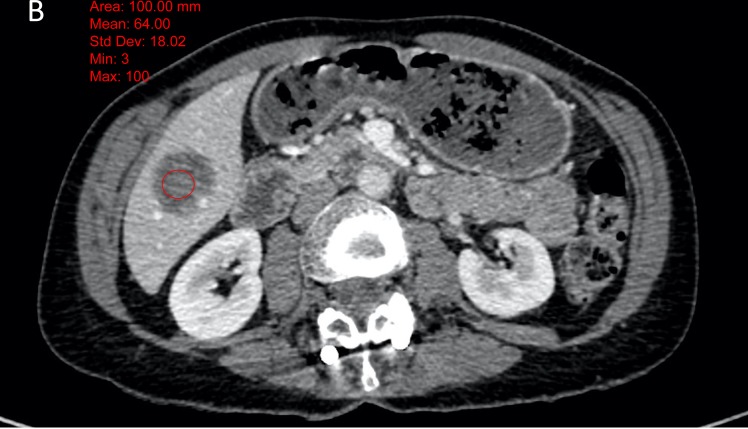

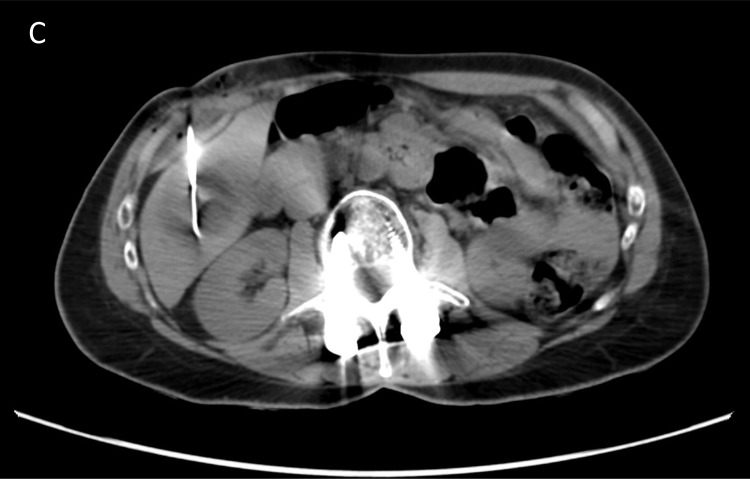
Example of 58 years old female patient, with a submaxillary gland cancer The attenuation of the metastatic lesion was measured as 27 HU on the non-enhanced image (fig A) and 64 HU on the enhanced images (figure B) (ΔHU = 37). The biopsy was a success with 65% of viable cells (figure C).

### Correlation between histology and neoplastic cellularity

For the whole population, semi-quantitative evaluation of necrosis was available in the pathology charts for 200 patients. Necrosis was graded 0 in 154 cases (77%), 1 in 22 cases (11%) and 2/3 in 24 cases (12%). There was a statistically significant association between graded necrosis and cellularity (mean cellularity = 52% ± 27.3, 66.6% ± 25.2 and 35.6% ± 27.5, in grade 0, 1 and 2/3, respectively, *P* < 0.001), but which did not reflect any trend.

### Causes of failures

The presence of necrosis was the etiology of the failure in ten cases (16%), while the presence of fibrosis was responsible of the failure in 23 (38%) cases. The remaining failures (28, 35%) were linked to absent or insufficient tumor (Table 4).

**Table 4:** Causes of failures

	Surgery	Palpation	Endoscopy	CT	Total
	*N* = 49	*N* = 28	*N* = 22	*N* = 122	*N* = 221^*^
**Number of failures**	6 (12%)	3 (11%)	12 (54%)	40 (33%)	61 (28%)
**Causes of failure**					
* No tumor	1 (16%)	2 (67%)	6 (50%)	7 (17.5%)	16 (26%)
* Insufficient tumor tissue	0 (0%)	0 (0%)	2 (17)	10 (25%)	12 (19%)
* Fibrosis (grade 2 or 3)	4 (66%)	1 (33%)	3 (25%)	15 (37.5%)	23 (38%)
* Necrosis (grade 2 or 3)	1 (16%)	0 (0%)	1 (8%)	8 (20%)	10 (16%)

* Technical issue with no biopsy in 5 patients, death before biopsy in 2 patients.

**Figure 4:**
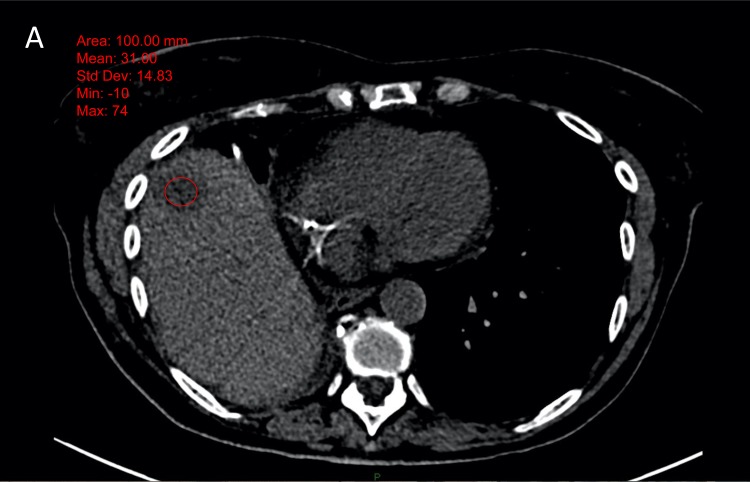

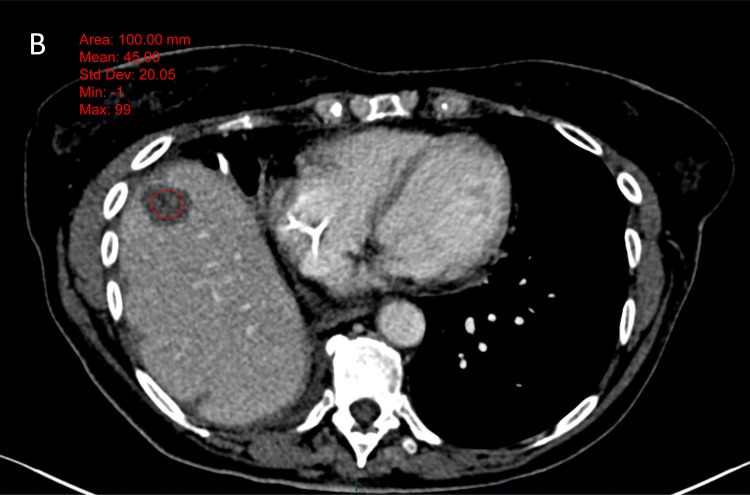

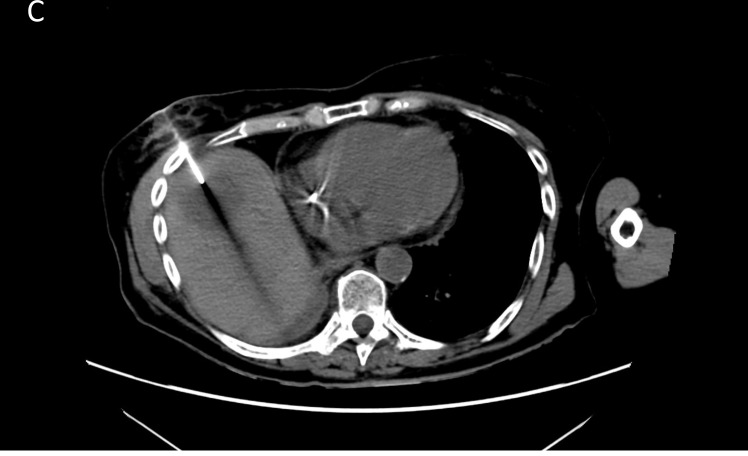
Example of 65 years old male patient, with a lung cancer The attenuation of the metastatic hepatic lesion was measured as 31 HU on the non-enhanced image (fig A) and 45 HU on the enhanced images (figure B) (ΔHU = 14). The biopsy was a failure with 20% of viable cells (figure C).

## DISCUSSION

In this study, we found that the prognostic factors of biopsy success were the absence of an ongoing chemotherapy, a lymph node or soft tissue location and higher mean differential attenuation (ΔHU) on CT. Moreover, biopsy failure was more frequent with CT guidance than with surgical or palpation-guided biopsy.

Molecular assessment requires a sufficient amount of neoplastic cells to reach the best efficacy in genetic alteration detection. There is a lack of consensus about the threshold of cellularity mandatory for biomolecular testing, which differs depending on the teams and the published studies. Some studies have chosen a tumor cell content threshold greater than 50% in order to qualify the biopsies for genomic testing [12], while other studies reported that DNA alteration (gains and loss) could be properly detected up to 20% tumor cells content [13]. For the SHIVA trial, samples containing > 50% of tumor cells were initially considered suitable for DNA extractions and genomic analyses. That threshold was switched to 30% after the feasibility part of the study. Neoplastic cellularity varies among tumors according to two ways, spontaneous heterogeneity of tumor tissue and post-therapeutic necrotic, fibrosis and cellular rearrangements due to multiple lines of chemotherapy or radiotherapy [14]. In the context of patients with refractory cancer, who get multiple lines of chemotherapy, the minimal mandatory amount of tumor cells could be difficult to obtain.

First, our study showed that the neoplastic cellularity was lower in patients who had ongoing chemotherapy during the biopsy, and consequently the rate of failure was higher in this subgroup of patients. This could be explained by the fact that chemotherapy induces cell death and decrease of tumor cells [15]. In the patients without ongoing chemotherapy, after the last chemotherapy course, the tumor could have grown and therefore the number of tumor cells increased. Surprisingly, there was no significant correlation between number of previous chemotherapy lines and neoplastic cellularity. Moreover, the tumor cellularity was higher in patients with > 5 lines of chemotherapy, which could be explained by a confounding factor. One of the hypotheses is that those lesions, with a high level of chemotherapy lines, were refractory to any treatment and contained a high content of viable tumor cells. However, Tacher et al tried in MOSCATO01 study to identify factors associated with cellularity in image-guided biopsy samples in any type of cancers as our study did. They found no correlation between recent chemotherapy defined as last administration treatment < 1 month and cellularity [18]. This difference is probably explained by the fact that 66% of our patients were under treatment at the time of the biopsy.

Second, we found that surgical and palpation-guided biopsies had the best results (success rate of biopsies, 90 and 88%, respectively). This result is not surprising since surgeons got larger samples of tissue in surgical biopsy, as they removed usually the entire lesion (lymph nodes, tumor recurrences…). Moreover, as well as for palpation-guided biopsies, the lesion was directly visualized and targeted. Indeed, data of phase III TRIBUTE study showed that small samples were more likely to fail for molecular profiling with a failure rate of 55 to 65% in small samples (bronchoscopic transbronchial needle aspiration and CT-guided biopsy) compared with 24% in larger excisional biopsy or resection specimen [16]. In our study bronchoscopy showed the lowest results, according to literature [20]. The CT-guided biopsies had intermediate results with 67% of success, between surgery/palpation biopsy and endoscopic biopsies.

Third, biopsies success occurred more often in lymph nodes (92%) and soft tissues (91%), and was the lowest in lung (bronchoscopy and CT-guided biopsies). It would explain, with the size of biopsy, the best performance of palpation and surgical biopsies compared to CT-biopsies where lymph nodes and soft tissues represent only 7 and 9% of the whole CT-guided biopsies, respectively. However, we don't have any explanation as to why success was higher in lymph nodes and soft tissue than in visceral metastasis, even in CT-guided biopsies. Interestingly, Tacher et al. also found that cellularity was higher in other sites (including lymph nodes and soft tissue) than in liver and lung [18].

Finally, the causes of failure in the pathological preparation were variable. On the one hand, 38 samples (45% of failures) in the whole population included no tumor or insufficient material. As expected, it concerned mostly CT-guided needle core biopsy and endoscopic biopsies. In the sub-group of CT-guided biopsies, 17 of the 122 CT-guided biopsies (14%) showed no tumor or insufficient tumor. For insufficient tumor tissue, this raises the question of the size of the needles used and the number of biopsy samples. No strong recommendation about needle size was established in the literature in this context. Indeed, results in several studies showed the feasibility of sufficient tissue acquisition for molecular profiling either using 18-gauge or 20-gauge true-cut biopsy needles *via* 17-gauge or 19-gauge coaxial needles, while showing no significant difference of complications [17, 21, 22]. The number of biopsy samples remains also questionable but results of the MOSCATO01 trial with an average of 6 samples showed better results than our study without a higher complication rate. On the other hand, the low success rates were linked to the presence of necrosis or fibrosis. However, no significant correlation between necrosis and cellularity was found as in the MOSCATO01 study [18], while the presence of fibrosis was more often responsible of failure than presence of necrosis. This reinforces the interest in intra-tumoral targeting in CT-guided biopsies, to identify and avoid necrosis and even further fibrosis. This could lead to realization of delayed enhanced CT acquisitions to better identify fibrosis area. In this context, functional imaging as PET-CT or diffusion-weighted MRI could be interesting and should be evaluated. However, to be efficient, an accurate registration is needed or MRI/PET-guided biopsy should be performed.

Our study had several limitations. First, more than half of the CT-guided biopsies were in liver (*n* = 63) and soft tissues (*n* = 11). These biopsies could have been guided by ultrasound, which is non-radiating and often allows for a shorter procedure than that under CT guidance, but we decided to use a technique permitting a retrospective analysis of images (precise size of tumor, measure of attenuation on non-enhanced and enhanced images). Second, our gene laboratory chose a threshold of 30% to define success of biopsy to perform molecular analysis, which is partly arbitrary; some teams chose a threshold of 50% [12], others showed that 20% was sufficient [13]. Third, we chose to take four samples of tumor tissue in each CT-guided biopsy, two for pathology department to evaluate necrosis and fibrosis, and two for the gene laboratory. Some teams [23] showed that results of biopsy could show some variation among samples of the same tissue, which suggests than multiplying the samples could increase the quantity of neoplastic cells. Fourth, the retrospective review of the presence of necrosis and/or fibrosis in the tissue samples concerned only the cases of failures and would introduce a bias. At last, we did not evaluate the performance of functional imaging (PET/CT, PET/MRI) to improve the targeting of biopsy. However, it was not the aim of the current study.

In conclusion, ongoing chemotherapy decreased tumor cell content and consequently the success of the biopsy samples for molecular profiling. Biopsy of soft tissues and lymph nodes seems to be more profitable than that of visceral metastasis, even in CT-guided biopsies.

## Abbreviations

BMI: Body Mass Index
CT: Computed-Tomography
DNA: Deoxyribo Nucleic Acid
ENT: Ear Nose Throat
HU: Hounsfield Unit
MRI: Magnetic Resonance Imaging
PACS: Picture Archiving and Communication System
PET-CT: Positron Emission Tomography-Computed Tomography
ROI: Region of interest
